# Differential Responses of Human Regulatory T Cells (Treg) and Effector T Cells to Rapamycin

**DOI:** 10.1371/journal.pone.0005994

**Published:** 2009-06-22

**Authors:** Laura Strauss, Malgorzata Czystowska, Marta Szajnik, Magis Mandapathil, Theresa L. Whiteside

**Affiliations:** Department of Pathology, University of Pittsburgh School of Medicine and University of Pittsburgh Cancer Institute, Pittsburgh, Pennsylvania, United States of America; New York University School of Medicine, United States of America

## Abstract

**Background:**

The immunosuppressive drug rapamycin (RAPA) promotes the expansion of CD4^+^ CD25^high^Foxp3^+^ regulatory T cells via mechanisms that remain unknown. Here, we studied expansion, IL-2R-γ chain signaling, survival pathways and resistance to apoptosis in human Treg responding to RAPA.

**Methodology/Principal Findings:**

CD4^+^CD25^+^ and CD4^+^CD25^neg^ T cells were isolated from PBMC of normal controls (n = 21) using AutoMACS. These T cell subsets were cultured in the presence of anti-CD3/CD28 antibodies and 1000 IU/mL IL-2 for 3 to 6 weeks. RAPA (1–100 nM) was added to half of the cultures. After harvest, the cell phenotype, signaling via the PI3K/mTOR and STAT pathways, expression of survival proteins and Annexin V binding were determined and compared to values obtained with freshly-separated CD4^+^CD25^high^ and CD4^+^CD25^neg^ T cells. Suppressor function was tested in co-cultures with autologous CFSE-labeled CD4^+^CD25^neg^ or CD8^+^CD25^neg^ T-cell responders. The frequency and suppressor activity of Treg were increased after culture of CD4^+^CD25^+^ T cells in the presence of 1–100 nM RAPA (p<0.001). RAPA-expanded Treg were largely CD4^+^CD25^high^Foxp3^+^ cells and were resistant to apoptosis, while CD4^+^CD25^neg^ T cells were sensitive. Only Treg upregulated anti-apoptotic and down-regulated pro-apoptotic proteins. Treg expressed higher levels of the PTEN protein than CD4^+^CD25^neg^ cells. Activated Treg±RAPA preferentially phosphorylated STAT5 and STAT3 and did not utilize the PI3K/mTOR pathway.

**Conclusions/Significance:**

RAPA favors Treg expansion and survival by differentially regulating signaling, proliferation and sensitivity to apoptosis of human effector T cells and Treg after TCR/IL-2 activation.

## Introduction

Naturally occurring CD4^+^CD25^high^Foxp3^+^ regulatory T cells (nTreg) are essential for maintaining tolerance to self and regulating immune responses to foreign antigens. Thus, Treg have been implicated in the pathogenesis of autoimmune diseases, transplant rejection and infectious diseases [1], [2]. However, Treg can also sabotage antigen-specific immune responses against tumors as well as microbes and have been proposed as a potential mechanism of immune suppression in patients with cancer or chronic HIV-1 infection [3]–[6].

Recent clinical studies suggest that in patients with autoimmune or allergic diseases, thymus-derived nTreg are reduced in numbers [7] and/or have reduced suppressive functions [8]–[10]. Therefore, their replacement with CD4^+^CD25^+^ Treg cells obtained from normal donors or *in vitro* expanded autologous functional CD4^+^CD25^+^ Treg represents a new paradigm for immunotherapy of autoimmune diseases. Recent studies in mice have shown that donor-type CD4^+^CD25^+^ Treg cells do not induce GVHD after major histocompatibility complex (MHC)-mismatched bone marrow transplantation but instead suppress GVHD induced by non-regulatory donor T cells [11], [12]. Importantly, a co-transfer of CD4^+^CD25^+^ Treg cells neither interfered with stem cell engraftment [11], [13] nor abrogated the beneficial anti-tumor activity of donor T-cell infusions [13], [14]. These murine studies are encouraging and provide a solid rationale for investigations of *in vitro* expansion strategies and adoptive transfer of human Treg cells for therapy of T-cell mediated autoimmune diseases or transplant rejection.

Discrimination of Treg from CD4^+^CD25^+^ effector non-Treg (conventional T cells or Tconv) and selective expansion of Treg on a clinical scale are limiting steps in the development of successful Treg-based immunotherapy. To date, no specific markers for human Treg have been identified and validated. Foxp3, a transcription factor considered by many as the Treg marker, appears to be also transiently expressed in activated Tconv [15]. We have depended on the isolation of CD4^+^CD25^high^ T-cell subset in our studies of human nTreg [16]. In contrast to murine cells, human CD25^high^ cells are not a clearly-defined, discrete population, because there exists a large and overlapping population of CD25^low/interm^ T cells. Thus, co-purification of these T cells with CD4^+^CD25^high^ Treg might be responsible for weak suppressor activity observed in studies of human CD4^+^CD25^+^ cells isolated from peripheral blood of normal donors [17]. Others reported that removal of CD49d^+^/CD127^+^ cells yields a population of Foxp3^+^ Treg which is free of contaminating CD25^+^ effector cells (18). Because CD4^+^CD25^high^ Treg represent a minor subset (<2%) of the peripheral CD4^+^ T cells in humans [16], [17], their *in vitro* expansion from peripheral blood for clinical use represents a challenge. To date, human Treg used in therapy have been obtained from the cord blood [19]–[21].

Approaches proposed for the elimination of non-Treg cells overgrowing in cultures of incompletely purified Treg include the use of the immunosuppressive drug rapamycin (sirolimus, RAPA). We and Battaglia et al have shown that RAPA selectively promotes expansion of human CD4^+^CD25^high^Foxp3^+^ T cells endowed with potent suppressor function in normal donors, patients with cancer or those with diabetes [16], [22], [23]. Though RAPA has been reported to induce Treg-dependent immunosuppression *in vitro* and *in vivo* [24]–[27], the exact mechanism of its action on Treg cells remains largely unknown. We have recently reported that one of the mechanisms through which RAPA can enrich for CD4^+^CD25^high^ Treg may be its ability to selectively induce apoptosis in TCR-activated CD4^+^CD25^+^ Tconv [16]. In contrast, activated nTreg which are constitutively CD25^high^Foxp3^high^ remain resistant to RAPA-mediated apoptosis [16]. These preliminary results suggest that although human Treg subsets may express many of the same surface markers or receptors and produce the same cytokines as conventional CD4^+^CD25^−^ or CD4^+^CD25^low^ T cells, proliferation of these T cell subsets in response to TCR-mediated signals and IL-2 might be differentially regulated. This divergent regulation via the distinct molecular pathways could be a key mechanism for maintaining the homeostatic balance between Treg and Tconv. Further, it could be exploited for selective *in vitro* or *in vivo* expansion of antigen-specific Treg or T effector cells for therapy of patients with T-cell mediated diseases.

In this manuscript, we first define optimal conditions for *ex vivo* expansion of human Treg in the presence of RAPA and then demonstrate differential effects of RAPA on Tconv and Treg with respect to the Bcl-2 family protein expression, utilization of the STAT vs. PI3K/mTOR pathways and the PTEN protein expression.

## Results

### Responses of Treg (CD4^+^CD25^+^Foxp3^+^) and conventional (CD4^+^CD25^neg^) T cells to RAPA in the presence of CD3/CD28 Ab crosslinking and IL-2

**Figure 1:**
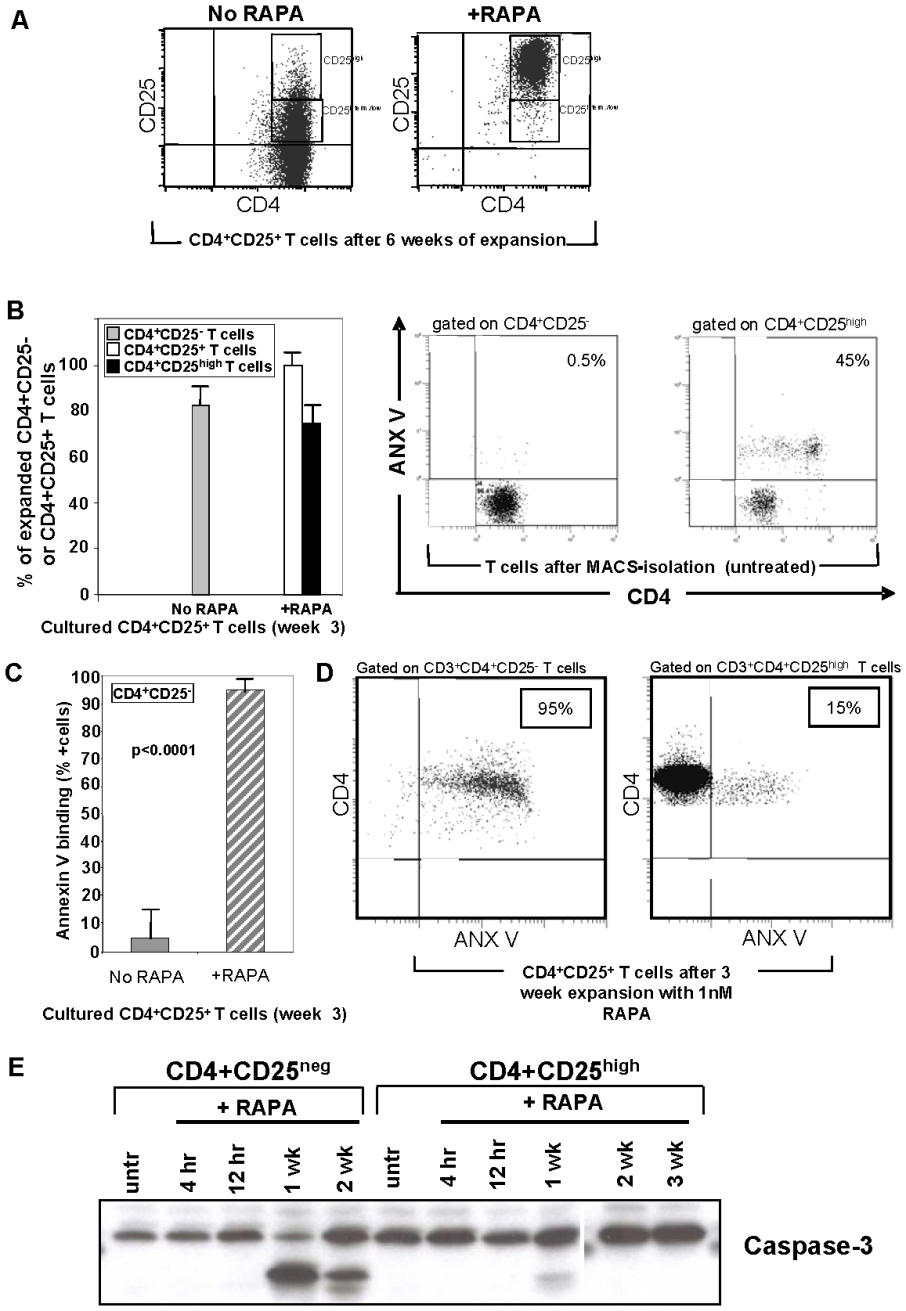
The phenotype and sensitivity to apoptosis of CD4^+^CD25^high^ T cells expanding±RAPA. A. Gating strategy used to designate CD25^high^ and CD25^inter/low^ T cells within the CD3^+^CD4^+^ T cell population. Note that after 6 weeks of culture+RAPA, nearly all cells are CD25^high^. B. Left panel, percentages of expanded CD4^+^CD25^neg^ or CD4^+^CD25^+^ T cells±RAPA after 3 weeks in culture. Data are means±SD cells in 10 cultures initiated with MACS-purified CD4^+^ T-cell subsets. Right panels, ANX V binding to fresh, uncultured MACS-isolated CD4^+^CD25^−^ and CD4^+^CD25^high^ T cells. A representative dot blot is shown. C. ANX V binding to CD4^+^CD25^−^ T cells in 3 week cultures±RAPA. The data are mean percentages±SD of ANX V^+^ cells in 10 cultures. Note that only few CD4^+^CD25^neg^ cells expanding in cultures with no RAPA bind ANX V, while all CD4^+^CD25^neg^ cells present in +RAPA cultures bind ANX V. D. ANX V binding to CD4^+^CD25^neg^ and CD4^+^CD25^high^ T cells after 3 weeks of culture+1 nM RAPA. A representative dot blot is shown. E. Western blots of CD4^+^CD25^high^ and CD4^+^CD25^neg^ T cells±RAPA showing caspase-3 activation only in the CD4^+^CD25^neg^ T-cell subset +RAPA.

Our initial experiments indicated that RAPA can selectively promotes expansion of CD4^+^CD25^high^Foxp3^+^ human Treg isolated from the peripheral circulation of normal donors and activated with anti-CD3/CD28 Ab+IL-2 [16]. The CD4^+^CD25^high^ T cell subset used in our experiments was previously shown to consist largely of Foxp3^+^ cells [3], [5], [16]. Cultured CD4^+^CD25^+^ cells down-regulated surface expression of CD25 in the absence of RAPA (R0) (Figure 1A, *left panel*). In contrast, the parallel RAPA-containing cultures (+R), were enriched in CD4^+^CD25^high^ T cells (Figure 1A, *right panel*). In the +R cultures, CD4^+^CD25^+^ and CD4^+^CD25^high^ T cells were the expanding cell populations. In contrast, R0 cultures largely contained proliferating CD4^+^CD25^neg^ T cells (Figure 1B *left panel*). These results suggested that RAPA inhibited expansion of CD4^+^CD25^neg^ cells while promoting proliferation of CD4^+^CD25^+^ T cells.

### Apoptosis of CD4^+^CD25^neg^ T cells in the presence of RAPA

To analyze whether RAPA inhibits T responder cell proliferation by selectively inducing apoptosis of CD4^+^CD25^neg^ T cells, we examined ANX V binding to CD4^+^CD25^+^ and CD4^+^CD25^neg^ T cells before and after their culture in the presence of RAPA for 3–6 weeks. Fresh MACS-separated CD4^+^CD25^neg^ cells failed to bind ANX V, while 45% of autologous CD4^+^CD25^+^ T cells were ANX V^+^ (Figure 1B , *right panels*). This result suggests that these CD4^+^CD25^+^ T cells were undergoing activation induced cell death (AICD). In contrast, ANX V binding to CD4^+^CD25^neg^ cells cultured in the presence of RAPA (+R) for 3 weeks was very high (90±10%), while significantly lower ANX V binding to CD4^+^CD25^neg^ cells (p<0.0001) was evident in proliferating R0 cultures (Figure 1C). As also shown in Figure 1D for a representative culture, significantly higher percentages of ANX V^+^ CD4^+^CD25^neg^ T cells were observed in 3 week +R cultures than of CD4^+^CD25^high^ T cells (95% vs. 15%). Analogous data were obtained when these cell subsets were cultured+R for 6 weeks (data not shown). ANX V binding to CD4^+^CD25^+^ and CD4^+^CD25^neg^ T cell subsets was similar when these cells were cultured without RAPA (23±14% vs. 20±15%; p<0.44; data not shown).

In addition to ANX V binding, caspase-3 cleavage into its active fragments was evaluated by Western blots in CD4^+^CD25^high^ and CD4^+^CD25^neg^ cell subsets +RAPA (Figure 1E). Caspase activity was observed only in CD4^+^CD25^neg^ cells +RAPA.

These results suggest that apoptosis-sensitive CD4^+^CD25^high^ T cells become resistant to apoptosis following TCR-mediated+IL-2 activation and subsequent culture in the presence of RAPA. In contrast, CD4^+^CD25^neg^ T cells become highly sensitive to apoptosis upon TCR and IL-2-mediated activation in the presence of RAPA and die.

### Proliferation kinetics of Treg and conventional T cells+RAPA

**Figure 2:**
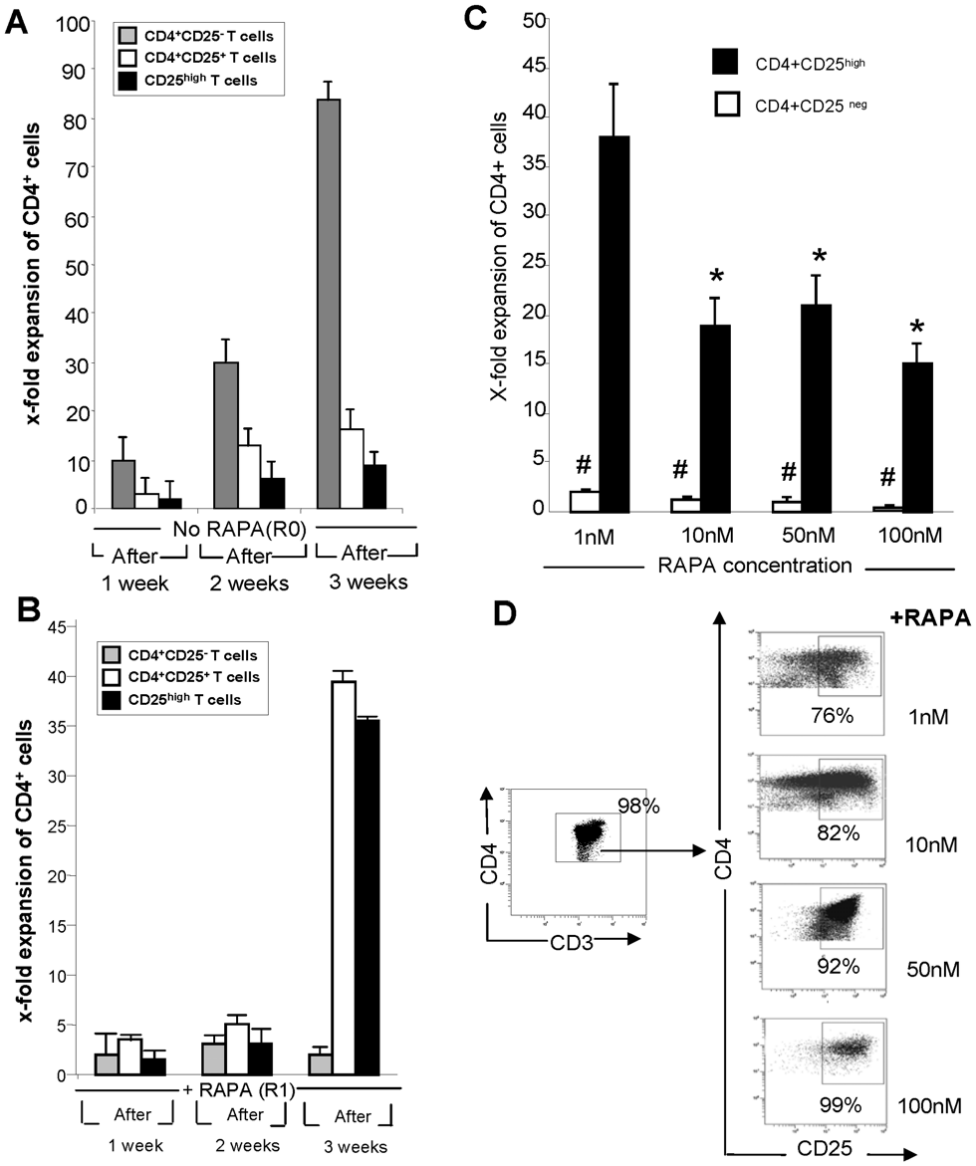
Expansion and suppressor activity of CD25^+^ T cells cultured in presence of rapamycin. A. Expansion rates of in vitro-expanded MACS purified human CD4^+^CD25^neg^, CD4^+^CD25^+^ and sorted CD4^+^CD25^high^ T cells cultured with irradiated feeder cells, anti-CD3/CD28 Ab-coated beads and 1,000 IU/mL of IL-2 in the absence of RAPA. Cell counts were performed at weekly intervals throughout culture period. The expansion rate is the ratio of absolute cell counts after culture vs. counts of the purified (by AutoMACS or sorting) cells before culture. The data are means±SD of experiments performed with cells of 10 NC. B. Results of the same experiments as in A but performed+1 nM RAPA. C. Expansion after culture for 3 weeks of CD4^+^CD25^+^ and CD4^+^CD25^neg^ T cells+RAPA used at various concentrations. The data are mean fold expansion±SD from 6 experiments with cells of different NC. Note the highest fold expansion of CD4^+^CD25^high^ T cells in the presence of 1 nM RAPA (p<0.001). Differences in expansion between CD4^+^CD25^high^ and CD4^+^CD25^neg^ T cells were also significant at p<0.0001. D. Enrichment of the cultures in CD4^+^CD25^high^ T cells at different concentrations of RAPA. The gating strategy was to include all CD4^+^ T cells in the gate, then to re-gate on CD4^+^CD25^high^ Treg. A representative dot blot of 5 experiments with cells of different NC is shown.

Next, the proliferation kinetics of autologous CD4^+^CD25^+^ and CD4^+^CD25^neg^ T cell subsets±RAPA were monitored by determining absolute cell numbers weekly during a 3-week expansion period (Figure 2A, B). The frequency of CD4^+^CD25^high^ T cells in these cultures was also determined by flow cytometry. CD4^+^CD25^neg^ T cells proliferated readily in the absence of RAPA, reaching a substantial fold expansion by week 3 (Figure 2A). CD4^+^CD25^neg^ cells did not proliferate in the presence of RAPA (Figure 2B). In contrast, both CD4^+^CD25^+^ and CD4^+^CD25^high^ T cells showed high expansion in the presence of RAPA but did not proliferate in its absence (Figure 2A). As CD4^+^CD25^+^ T cell subset consists largely of CD4^+^CD25^high^ Treg (see Figure 2D), the expansion rate of both these subsets is comparable in the presence of RAPA. Expansion of CD4^+^CD25^+^ T cells in +R cultures was delayed. This latency period might reflect the paucity of factors necessary for their proliferation, despite the presence of feeder cells. However, by week 3, sustained proliferation of CD4^+^CD25^+^ and CD4^+^CD25^high^, but not CD4^+^CD25^neg^ T cells, occurred, leading to significant expansion of the CD25^+^ subsets (Figure 2B). These findings together with the observation that RAPA induces apoptosis of nearly all activated CD4^+^CD25^neg^ T cells suggest that RAPA selectively promotes expansion of Treg present in PBMC by inducing cell death of “contaminating” CD4^+^CD25^neg^ Tconv.

To determine the optimal concentration of RAPA for expansion of single cell-sorted CD4^+^CD25^high^ Treg, we set up cultures containing RAPA at concentrations ranging from 1 to 100 nM. Figure 2C shows that CD4^+^CD25^high^ T cells obtained from the blood of six different NC proliferated best at the concentration of 1 nM of RAPA relative to Treg in cultures with higher RAPA concentrations. Figure S1 shows representative flow cytometry results for fresh and RAPA-expanded Treg. When the percentages of CD4^+^CD25^high^ cells were measured within the CD3^+^CD4^+^ subset in the RAPA-containing cultures after 3 weeks of incubation, the highest Treg purity (99%) was obtained in cultures containing 100 nM of RAPA (Figure 2D). 1 nM RAPA cultures contained a mean of 76%±4 (SD) of CD4^+^CD25^high^ Treg (Table 1).

**Table 1:** Enrichment in CD4^+^CD25^high^ Treg in cultures containing various RAPA concentrations^a^.

	% CD4^+^CD25^high^ T cells	
	range	mean±SD
No RAPA	16–22	19±8
+ RAPA 1 nM	71–79	76±4
+ RAPA 10 nM	76–86	81±6
+ RAPA 50 nM	85–95	87±6
+ RAPA 100 nM	91–99	96±9

a The data are results of flow cytometry analyses using 3 week cultures of CD4^+^CD25^high^ T cells obtained from 6 subjects and expanded in the presence or absence of RAPA. The gate was set on the CD4^+^CD25^high^ subset of cultured cells.

### Phenotypic characteristics of Treg expanded by RAPA

**Figure 3:**
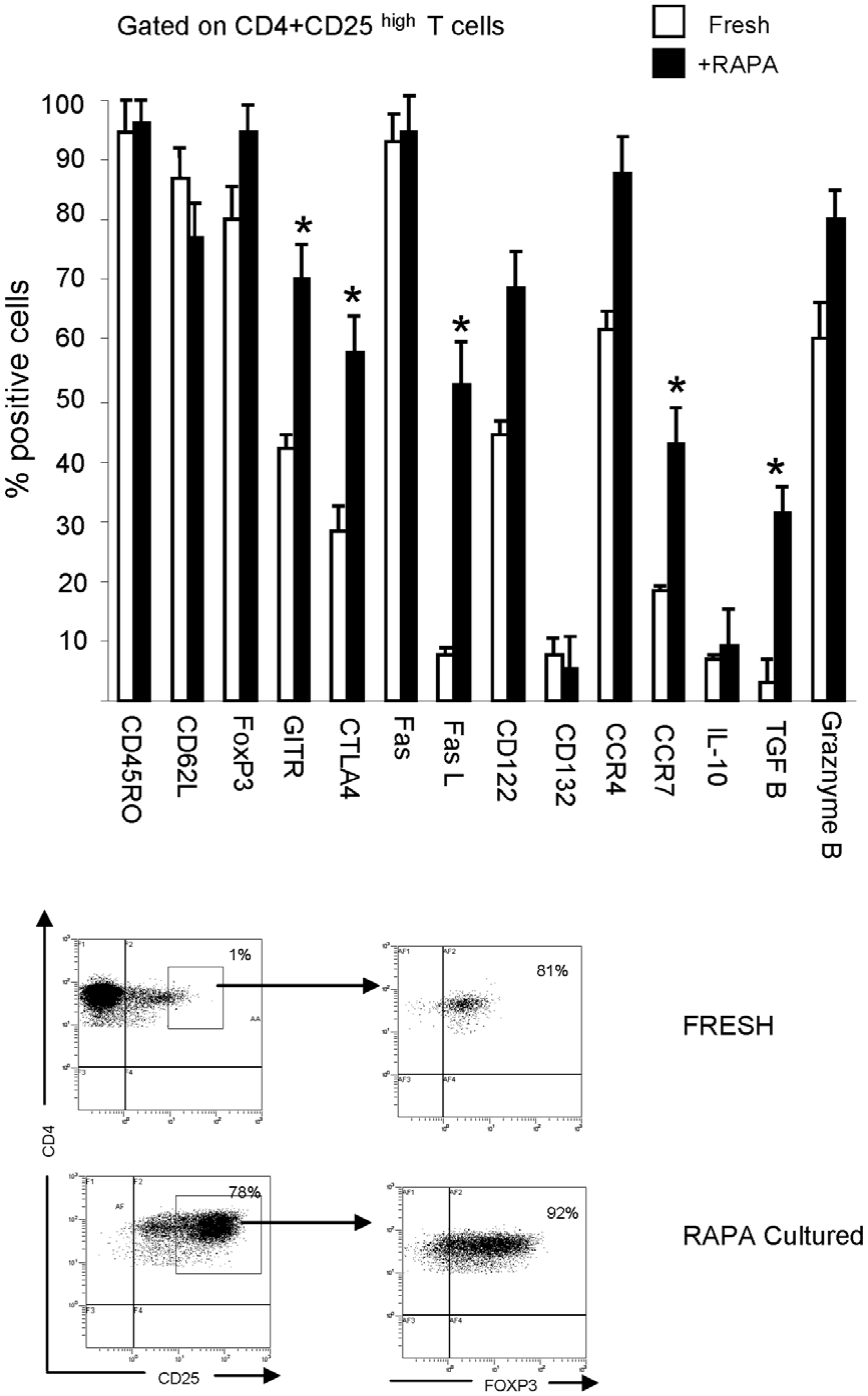
Phenotypic analysis of CD4^+^CD25^+^ T cells cultured±1 nM RAPA. A. MACS-purified CD4^+^CD25^+^ cells were cultured for 3 weeks, and then their phenotype was assessed by flow cytometry. Culture conditions±1 nM RAPA were as described in Materials and Methods. Freshly-isolated CD4^+^CD25^high^ T cells were similarly phenotyped. The flow cytometry data, obtained by gating on CD4^+^CD25^high^ T cells in fresh PBMC or+RAPA cultures, are mean percentages±SD from experiments with cells of 6 NC. The asterisks indicate significant differences at p<0.001 in the percentage of positive cells relative to freshly isolated Treg.

The phenotype of CD4^+^CD25^+^ T cells expanded in +R cultures was significantly different from that of T cells outgrown in R0 cultures (data not shown). The latter were CD4^+^CD25^neg^ and did not express Foxp3, CTLA-4 or GITR. On the other hand, the CD4^+^CD25^high^ T cells expanded in +R cultures had the memory phenotype (CD45RO^+^(RA^−^)) and expressed the characteristic Treg markers, including Foxp3, CD62L, CTLA4 and GITR (Figure 3). These cells were strongly positive for Fas and FasL, and showed expression of granzymes and TGF-β. Their phenotype was compared to that of freshly harvested, FACS-sorted CD4^+^CD25^high^ cells, and the gating used in these experiments is illustrated in Figure 3, with representative dot plots shown in Figure S1. Several differences were identified between fresh and RAPA-expanded Treg (Figure 3). A higher proportion of +R cells than fresh CD4^+^CD25^high^ Treg expressed TGFβ1 and the chemokine receptors CCR4 and CCR7. Nearly all fresh CD4^+^CD25^high^ T cells were Foxp3^+^, and 100% of the cells in all +R cultures remained Foxp3^+^. No differences in this phenotypic profile occurred in cells cultured for 3 or 6 weeks (data not shown). There were no significant differences in the Treg phenotypic profile in +R cultures established at RAPA concentrations ranging from 1 to 100 nM of the drug (data not shown). Overall, T cells expanded +RAPA had the phenotype that resembled that of Treg present in the peripheral circulation of NC, as previously reported by us [16].

### Suppressor functions of Treg expanded by RAPA

**Figure 4:**
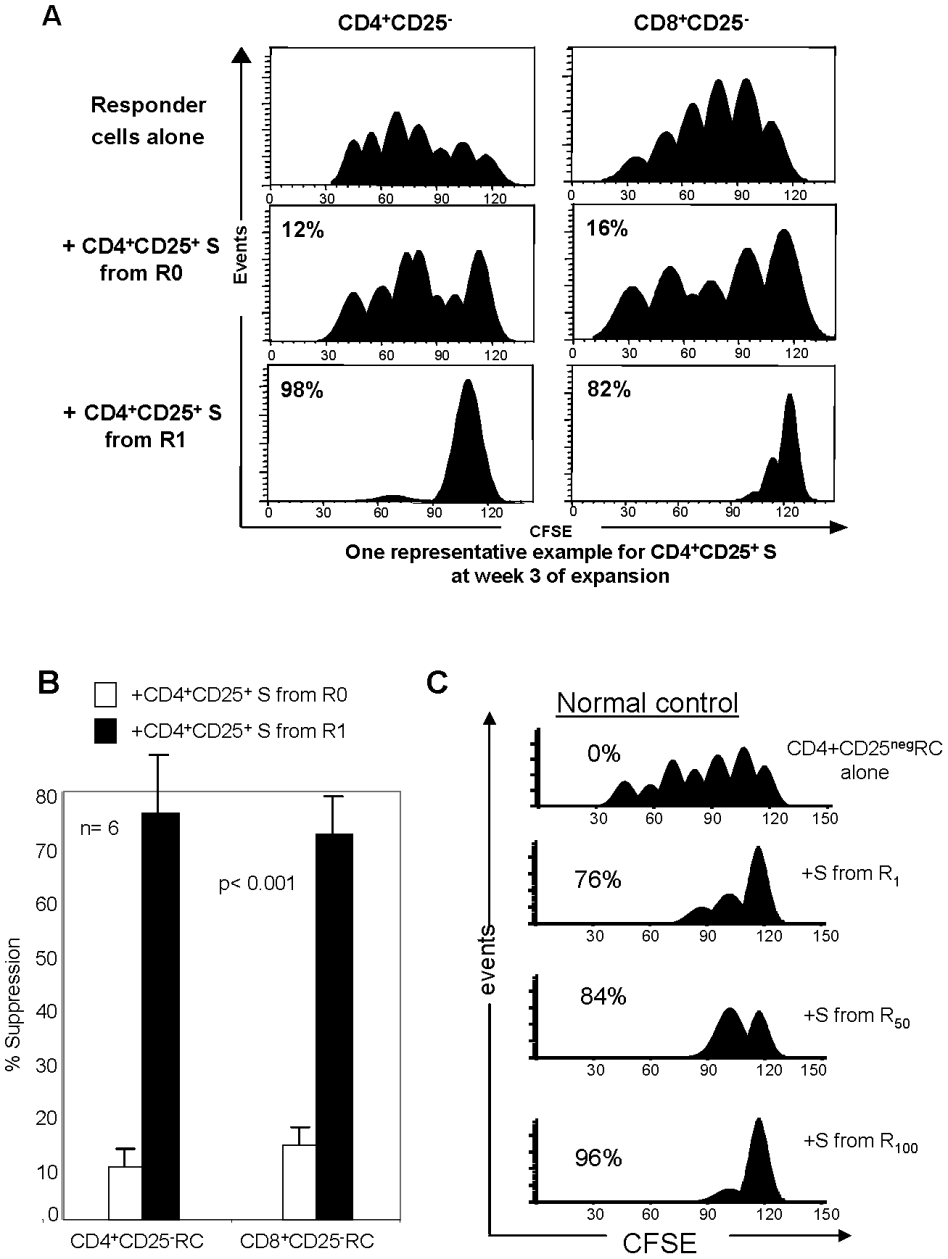
Suppression of proliferation of CD4^+^CD25^neg^ or CD8^+^CD25^neg^ responder cells with T cells cultured±1 nM RAPA. A. Fresh MACS-purified CD4^+^CD25^neg^ or CD8^+^CD25^neg^ T cells were used as CFSE-labeled responders. Autologous MACS-isolated CD4^+^CD25^+^ T cells cultured in presence of anti-CD3/CD28 beads and 1,000 IU/mL IL-2 in the presence or absence of 1 nM RAPA for 3 weeks were added at the 1∶1 ratio to responder cells. Gates were set on CD4^+^ and CFSE^+^ cells and analyzed using the ModFit program as described in Materials and Methods. Suppression of responder cell proliferation is indicated as %. A representative experiment of 10 performed is shown. B. The mean percentages±SD of proliferation suppression in CD4^+^CD25^neg^ or CD8^+^CD25^neg^ responder cells by suppressor cells generated in 3 week cultures±1 nM RAPA (n = 10). The p value is for differences between suppression mediated by S from R0 vs. S from R1 cultures. C. Suppression levels mediated by Treg expanded with different concentrations of RAPA. A representative experiment of 6 performed with cells of different NC is shown.

To evaluate suppressive activity of CD4^+^CD25^+^ T cells cultured±RAPA for 3 or 6 weeks, CFSE co-culture assays were performed. Suppressor (S) T cells (Treg) derived from the R0 or +R cultures were co-incubated with CFSE-labeled fresh autologous CD4^+^CD25^neg^ or CD8^+^CD25^neg^ responder T cells (ratio 1S∶1RC) stimulated with OKT3 and anti-CD28 Abs in the presence of 150 IU/mL of IL-2. Nearly complete suppression of RC proliferation was mediated by CD4^+^CD25^+^ Treg cells derived from 3 week cultures +RAPA (Figure 4A). Significantly weaker suppression (p<0.001) was mediated by Treg cells originating from R0 cultures (Figure 4B and Table 2). The mean % suppression obtained in CFSE-based suppression assays for all the R0 and +R cultures is shown in Figure 4B, while suppression mediated by CD4^+^CD25^+^ Treg cells of six NC cultured in the presence of various concentrations of RAPA is shown in Table 2. Suppression of RC proliferation was nearly complete with Treg cells cultured with 100 nM RAPA. Treg cells cultured in 1 nM RAPA also mediated high levels of suppression, as shown in Figure 4C and Table 3).

**Table 2:** Suppressor activity of Treg in R0 and R cultures^a^.

Suppressor cells	Responder cells	% suppression of proliferation	p value^b^
R0	CD4^+^CD25^neg^	10±2.5	
	CD8^+^CD25^neg^	18±9	
R	CD4^+^CD25^neg^	80±6	p<0.0001
	CD8^+^CD25^neg^	96±5	p<0.001

a The data are means±SD of percent suppression measured in co-cultures of proliferating autologous responder cells with Treg generated in R0 or R cultures. R cultures were set up with 1 nM RAPA. Responder cells were either CD4^+^CD25^neg^ or CD8^+^CD25^neg^. The co-cultures were established with cells obtained from 10 NC. The responder cell to Treg ratios were 1∶1 in all cultures.

b The p values are for the differences in the % suppression between Treg in the R0 and R cultures.

**Table 3:** Suppression of CD4^+^CD25^−^ cell proliferation by CD4^+^CD25^high^ T cells expanded in the presence of RAPA^a^.

	% suppression (mean±SD)
No RAPA	10±3
RAPA 1 nM	77±6
RAPA 50 nM	84±5
RAPA 100 nM	96±6

a Suppression of autologous responder cell proliferation with cells of 6 normal donors. Treg and responder cells were obtained from PBMC and cultured±RAPA as described in Materials and Methods.

Our results showed that CD4^+^CD25^neg^ Tconv expanded in the absence of RAPA (R0) contained few functional suppressor Treg. When CD4^+^CD25^neg^ T cells were co-cultured with autologous RC as controls in the presence of RAPA, they neither proliferated nor mediated suppression in CFSE assays. Thus, RAPA did not induce Treg in the CD4^+^CD25^neg^ T cell subset (data not shown).

### Expression of pro- and anti-apoptotic proteins in fresh and cultured T-cell subsets

Our results indicated that the CD4^+^CD25^high^ Treg generated in +R cultures mediated potent suppression and were resistant to spontaneous and activation-induced apoptosis. Apoptosis-sensitive CD4^+^CD25^neg^ T cells were eliminated in these cultures. To determine whether survival of TCR-activated Treg in the presence of RAPA was dependent on the BcL-2 family members, expression of Bcl-2, Bcl-xL and Bax proteins in T cells was analyzed by flow cytometry in the parallel +R and R0 cultures.

**Figure 5:**
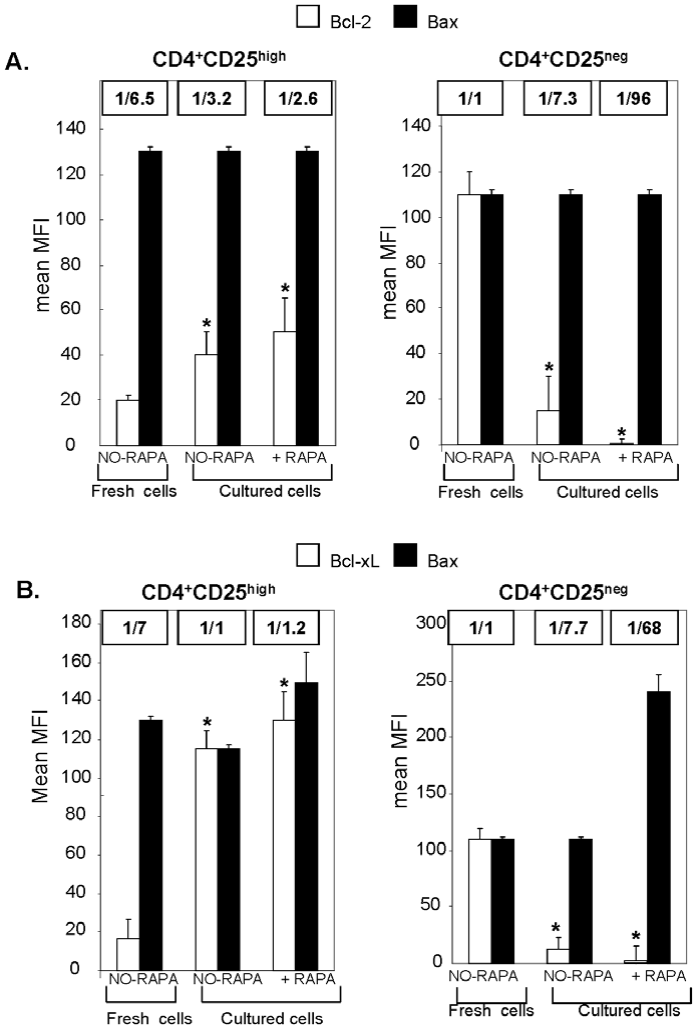
Expression levels the anti-apoptotic (Bcl-2 and Bcl-xL) and the pro-apoptotic (Bax) proteins in CD4^+^CD25^+^ or CD4^+^CD25^neg^ T cells measured before and after culture in the presence or absence of RAPA. The data are mean fluorescence intensity (MFI) mean values (±SD) for Bcl-2, Bcl-xL and Bax expression levels obtained from measurements with freshly isolated T-cell subsets or T-cell subsets cultured with RAPA or without RAPA. The ratios of Bcl-2/Bax or Bcl-xL/Bax are above the histograms. T cell subsets were separated by MACS from PBMC of 10 NC and cultured as described in Materials and Methods.

Expression levels (measured as MFI) of pro-apoptotic Bax and anti-apoptotic Bcl-xL or Bcl-2 were equal in the apoptosis-resistant fresh CD4^+^CD25^neg^ T-cells (Figure 5). However, in the apoptosis-sensitive fresh CD4^+^CD25^high^ T cells, expression levels of Bcl-2 and Bcl-xL were significantly lower than those of Bax, with the Bcl-2/Bax and Bcl-xL/Bax ratios of 1/6.5 to 1/7.3, respectively (Figure 5). After culture in the presence of RAPA for 3 weeks, CD4^+^CD25^neg^ cells were strongly positive for Bax but lost Bcl-2 expression, with the Bcl-2/Bax and Bcl-xL/Bax ratios of 1/96 and 1/68, respectively. In contrast, in the CD4^+^CD25^high^ T cells cultured with RAPA, the expression levels of anti-apoptotic Bcl-2 and Bcl-xL increased significantly (p<0.001), resulting in the Bcl-2/Bax and Bcl-xL/Bax ratios of 1/2.6 and 1/1.2, respectively (Figure 5). Similar data were obtained for the FLIP/Bax ratios (data not shown). In aggregate, these results suggest that RAPA up-regulated expression of the survival molecules in Treg, while in CD4^+^CD25^neg^ T cells it up-regulated expression of the pro-apoptotic molecules. The data support the hypothesis that RAPA-induced apoptosis may be the mechanism responsible for a demise of CD4^+^CD25^neg^ T cells and concomitantly for the selective outgrowth of CD4^+^CD25^+^ Treg in the presence of this drug.

### STAT3 and STAT5 signaling in CD4^+^CD25^neg^ and CD4^+^CD25^high^ T cells±RAPA

**Figure 6:**
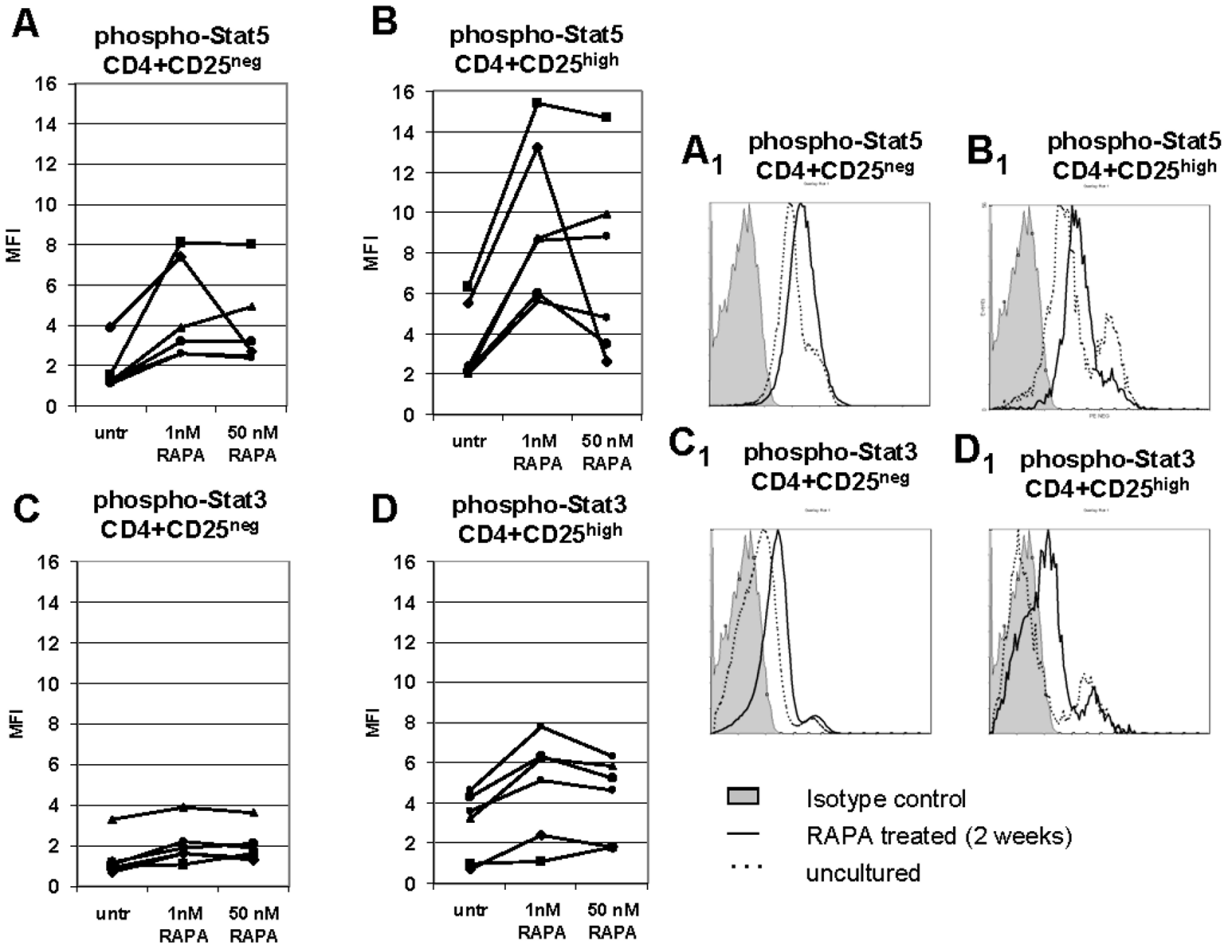
Activation of STAT5/3 proteins in CD4^+^CD25^neg^ and CD4^+^CD25^high^ T cells cultured at different RAPA concentrations. Mean fluorescence intensity (MFI) of phosphorylated STAT5 in CD4^+^CD25^neg^ and CD4^+^CD25^high^ T cells in A, and of phosphorylated STAT3 in B. T cells were tested by flow cytometry immediately after isolation and after short-term (4 h) culture with different RAPA concentrations (1 nM or 50 nM). Before analysis, the cells were briefly stimulated with 150 IU/mL IL-2 (A and B) or with 150 IU/mL IL-6 (C and D) and stained for phosphorylated STAT5 or phosphorylated STAT3, respectively. The gate was set on CD3^+^CD4^+^CD25^neg^ or CD3^+^CD4^+^CD25^high^ cells, respectively. Results obtained with T cells of 6 different NC are shown.

To further evaluate the functional status of freshly-harvested or RAPA expanded CD4^+^CD25^neg^ or CD4^+^CD25^high^ T cells, levels of phosphorylated STAT5 and STAT3 were measured in these T cells following short-term activation with cytokines. Single-cell sorted CD4^+^CD25^neg^ or CD4^+^CD25^+^ cells obtained from 6 NC were incubated±RAPA, stimulated with the relevant cytokines (150 IU/mL) for 20 min, permeabilized and treated with phosphorylated (phospho) STAT5 or STAT3 Abs. At baseline (no RAPA), phospho-STAT5 levels were higher in CD4^+^CD25^high^ T cells than in CD4^+^CD25^neg^ T cells (Figure 6A). The levels of phospho-STAT5 were up-regulated upon 4 h incubation+1 nM RAPA in CD4^+^CD25^high^ T cells of all 6 NC but only moderately in CD4^+^CD25^neg^ cells of 2/6 NC. At the higher RAPA concentration (50 nM) phospho-STAT5 levels tended to decline. At baseline, phospho-STAT3 levels were low in CD4^+^CD25^high^ cells of 2/6 donors and in CD4^+^CD25^neg^ cells of 5/6 donors. While incubation of CD4^+^CD25^high^ cells with 1 nM RAPA increased phospho-STAT3 expression in 5/6 donors, this up-regulation was much less pronounced than that of STAT5 (compare Figures 6B and D). These data are consistent with the hypothesis that activation of human Treg in the presence of RAPA is mainly STAT5 dependent.

**Figure 7:**
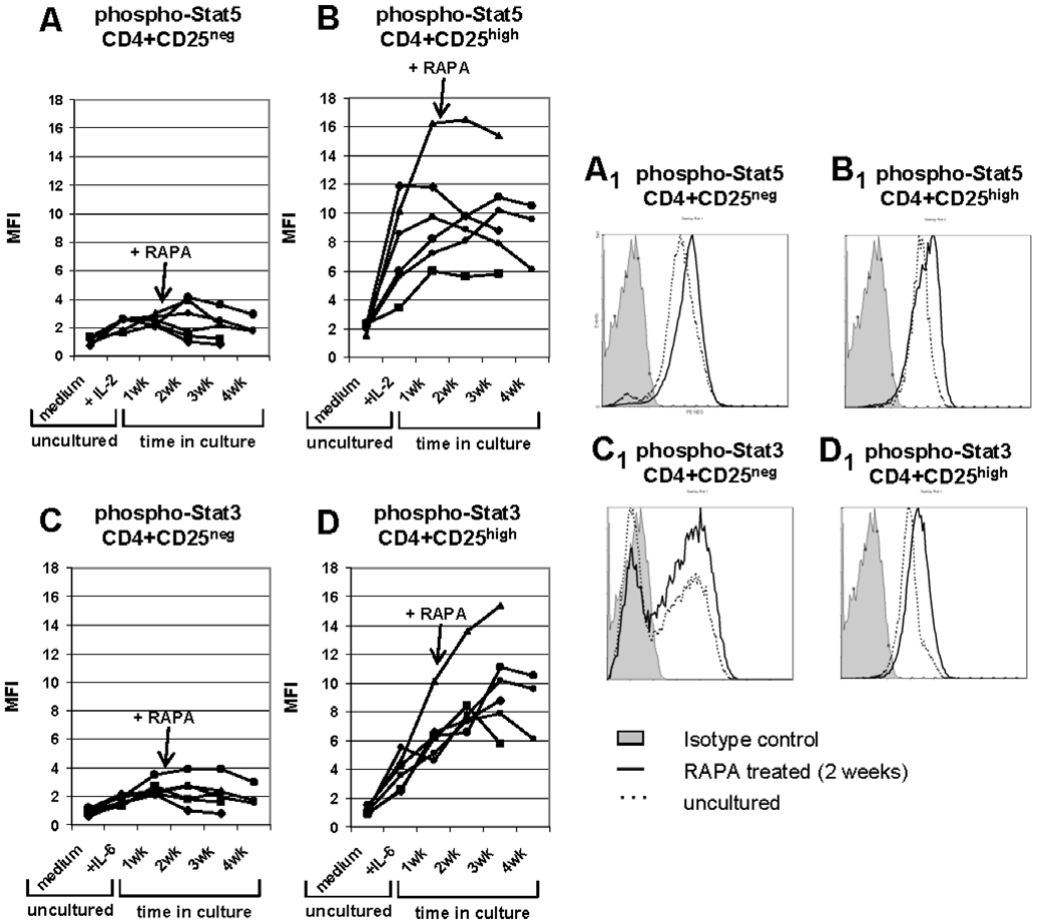
Activation of STAT5/3 proteins in fresh (untreated) and RAPA-expanded CD4^+^CD25^neg^ and CD4^+^CD25^high^ T cells. Mean fluorescence intensity (MFI) of phosphorylated STAT5 in CD4^+^CD25^neg^ in A, and CD4^+^CD25^high^ T cells in B and of phosphorylated STAT3 in CD4^+^CD25^neg^ in C and CD4^+^CD25^high^ T cells in D. PBMC-derived T cells were sorted into CD4^+^CD25^neg^ and CD4^+^CD25^+^ populations and tested by flow cytometry immediately after isolation and after culture in the presence of 1 nM RAPA (at baseline unstimulated, in medium) or were briefly stimulated with 150 IU/mL IL-2 (A and B) or with 150 IU/mL IL-6 (C and D) just before analysis. In addition, T cells were cultured for the indicated periods of time and stimulated with IL-2 and IL-6 just before flow cytometry analysis. The gate was set on CD3^+^CD4^+^CD25^neg^ or CD3^+^CD4^+^CD25^high^ T cells, respectively. RAPA was added to T-cell cultures at the indicated concentrations. Data obtained with T cells of 6 NC are shown. Fold expansion and percent suppression mediated by these cells are shown in Table 4.

To compare RAPA effects on STAT signaling in CD4^+^CD25^neg^ and CD4^+^CD25^high^ T cells cultured+1 nM RAPA, we measured phospho-STAT5 and phospho-STAT3 levels following 20 min stimulation of the cells with the relevant cytokines at baseline and at different time points in culture. As expected, the levels of phospho-STAT5 were considerably higher in CD4^+^CD25^high^ than in CD4^+^CD25^neg^ T cells following stimulation with IL-2 alone (Figures 7A and B). Upon RAPA addition and 1, 2 or 3 weeks in culture, CD4^+^CD25^high^ cells maintained high levels of STAT5 signaling. In contrast, in CD4^+^CD25^neg^ T cells, initial IL-2 stimulation failed to up-regulate phospho-STAT5 and upon RAPA addition, it remained at the baseline level. The high level of STAT5 phosphorylation correlated with increased fold expansion of the CD4^+^CD25^high^ T cells and their high suppressor function relative to those of CD4^+^CD25^neg^ T cells (Table 4). The addition of RAPA had a more dramatic effect on STAT3 signaling in CD4^+^CD25^high^ cells, as levels of phospho-STAT3 increased throughout the culture period (Figure 7D). No such increase was seen in CD4^+^CD25^neg^ cells +RAPA (Figure 7C). These signalling data are correlated to proliferation and suppression activity of the relevant cell subsets in Table 4.

**Table 4:** Treg cultured in the presence of RAPA up-regulated phospho-STAT5 as well as phospho-STAT3, had higher expansion rates and mediated higher suppression of autologous T responder cells than Tconv^a^.

	CD4^+^CD25^high^		CD4^+^CD25^neg^	
	no RAPA	+ RAPA	no RAPA	+ RAPA
Fold proliferation	6±2	39±9*	75±11*	±3
% suppression	11±2	78±7*	0	7±2

a The proliferation fold and percent suppression (at the 1∶1 ratio of Treg to RC) observed in 3 week cultures of Treg obtained from the 6 NC, whose Treg and Tconv were tested for phospho-STAT3 and phospho-STAT5 as shown in Figure 7. The data are mean percentages±SD. Asterisks indicate significant differences between no RAPA and+RAPA cultures at p<0.001.

Overall, the above data indicate that RAPA-driven proliferation of CD4^+^CD25^high^ Treg is dependent on the STAT pathway. In contrast, CD4^+^CD25^neg^ T cells did not signal via STATs whether in the presence or absence of RAPA.

### Elevated PTEN expression in RAPA-expanded CD4^+^CD25^high^ cells

**Figure 8:**
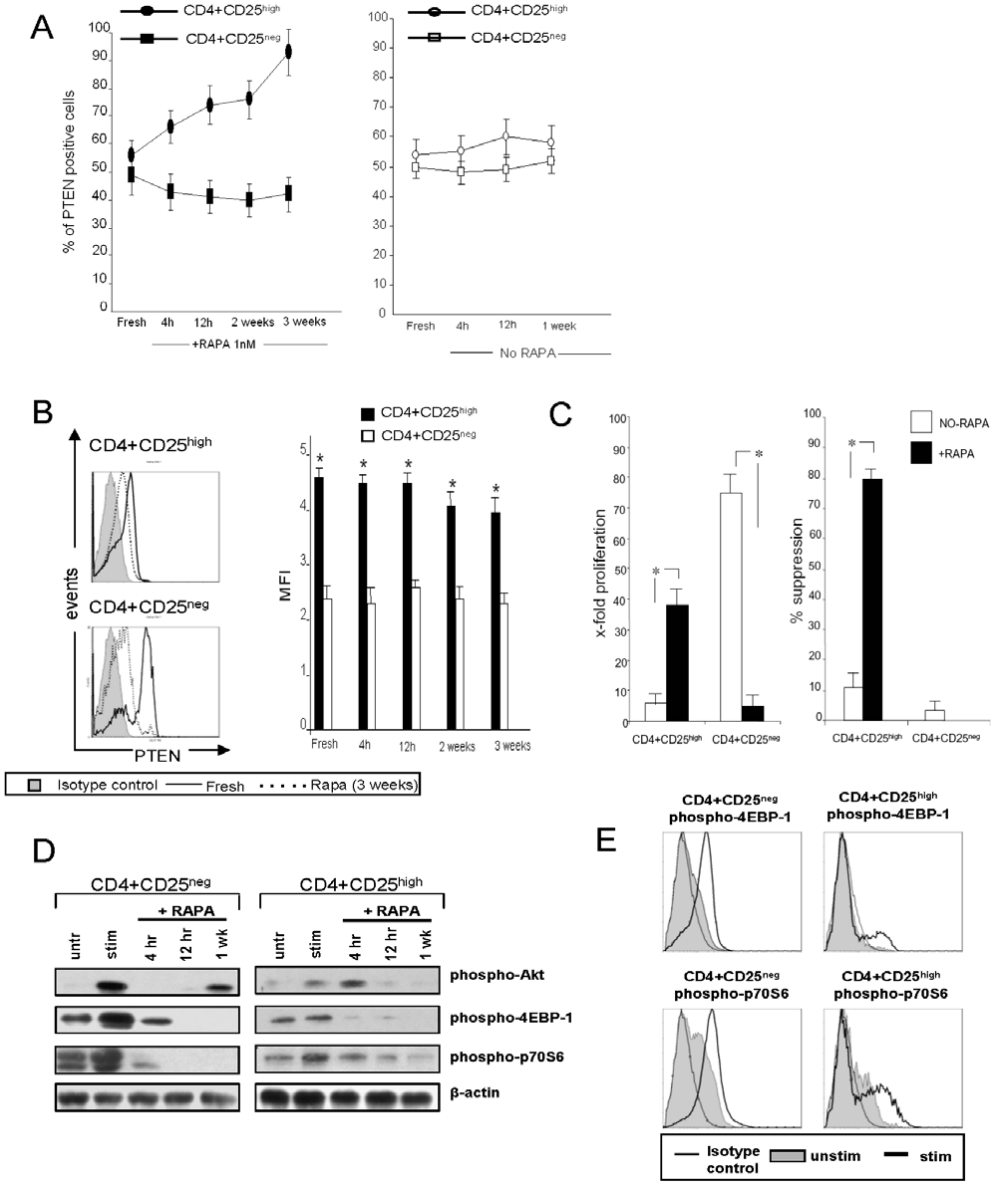
PTEN expression and signaling in fresh and RAPA-expanded CD4^+^CD25^high^ and CD4^+^CD25^neg^ T cells. A. Percentages of PTEN^+^ cells tested fresh (no RAPA) or after culture +RAPA for the indicated time periods. B. The MFI for PTEN expression in fresh and RAPA-expanded T cell cultures. C. Fold expansion and % suppression mediated by the same CD4^+^CD25^high^ and CD4^+^CD25^high^ T cell subsets cultured±RAPA on week 3. The data in A–C are means±SD from 6 experiments with cells of normal donors. Asterisks indicate significant differences between CD4^+^CD25^high^ and CD4^+^CD25^neg^ cells at p<0.001. C. Results of Western blots comparing expression levels of phospho-Akt, phospho-4EBP-1 and phospho-p70S6 in CD4^+^CD25^high^ and CD4^+^CD25^neg^ T cell subsets±RAPA. T cells were either unstimulated or stimulated with bead-coated anti-CD3/CD28 Abs and 100 IU/mL of IL-2 for 2 h and cultured in the presence of RAPA for the indicated time periods. The cells were harvested and used for Western blots. D. Flow cytometry histograms showing expression of phospho-4EBP-1 and phospho-p70S6 in freshly-isolated CD4^+^CD25^high^ and CD4^+^CD25^neg^ T cells subsets. Cells were stimulated as described above for 24 h and cultured in the presence of RAPA. They were harvested, permeabilized, stained and examined by flow ctyometry. Results are from one out of 3 experiments performed.

PTEN is a known negative regulator of the PI3K/mTOR pathway [30]. It has been reported that in mice, high PTEN levels are responsible for resistance of Treg to RAPA-induced apoptosis [31]. Therefore, we studied PTEN expression levels in human SCS CD4^+^CD25^high^ and conventional CD4^+^CD25^neg^ T cells±RAPA, with the expectation that the former will express high levels of PTEN. About half of freshly isolated CD4^+^CD25^high^ and CD4^+^CD25^neg^ T cells expressed PTEN. In cultures +RAPA, the percent of PTEN^+^CD4^+^CD25^high^ T cells gradually increased to reach close to 100% in 3 week cultures (Figure 8A). The MFI was also significantly higher in fresh CD4^+^CD25^high^ T cells, and in culture, the high level of PTEN expression (MFI) remained significantly higher than that in cultured CD4^+^CD25^neg^ cells (Figure 8C). In CD4^+^CD25^neg^ T-cell cultures +RAPA, PTEN^+^ cells declined in the frequency overtime (Figure 8A). The proliferation and suppression data for the CD4^+^CD25^high^ and CD4^+^CD25^neg^ T cell subsets used in these experiments are shown in Figure 8B. These data are consistent with the hypothesis that a high level of PTEN expression in Treg is important for their resistance to RAPA-induced cell death.

### PI3K/Akt/mTOR signalling in Treg

High levels of PTEN seen in Treg suggest that this survival pathway may be blocked in these T cells. To further examine this possibility, we studied phospho-Akt mTOR, phosphorylated mTOR expression and downstream phosphorylation of 4E-PB1 and p70S 6K1 in CD4^+^CD25^high^ and CD4^+^CD25^neg^ T cells. Western blot analyses showed that mTOR protein levels were comparable in the freshly-isolated T-cell subsets and remained unchanged in the presence of RAPA (data not shown). Phosphorylation of Akt, upstream of mTOR, was negligible in fresh CD4^+^CD25^high^ T cells relative to that in CD4^+^CD25^neg^ T cells (Figure 8D). When phosphorylation of the downstream targets, 4E-BP1 and p70S6K1, was examined in Western blots, low or no expression of the phosphorylated proteins was evident in fresh CD4^+^CD25^high^ T cells. In contrast, their expression was high in fresh CD4^+^CD25^neg^ T cells as was that of phospho-Akt. By flow cytometry (Figure 8E), the baseline expression of phosphorylated 4E-BP1 and p70S6K1 proteins was higher in the CD4^+^CD25^neg^ T cells than the CD4^+^CD25^high^ cells, and after OKT3 and IL-2 stimulation, it was substantially upregulated only in the CD4^+^CD25^neg^ cells. In RAPA-containing cultures of these cells, the expression of phosphorylated proteins in both the T cell subsets was blocked (Figure 7D). The data are consistent with the model suggesting that Treg do not utilize the PI3K/mTOR pathway, because it is down-regulated by PTEN, while proliferation and survival of CD4^+^CD25^neg^ conventional T cells depend on this pathway and are inhibited by RAPA.

## Discussion

For many years, RAPA (sirolimus) has been used as an immunosuppressive drug that induces peripheral tolerance, although the mechanism of its action is still not completely understood. Specifically, little is known about the effects of RAPA on expansion, function and survival of Treg subsets in man, although *in vitro* and *in vivo* studies of murine Treg have provided insights into molecular pathways that regulate Treg expansion [reviewed in 32]. We and others have reported that RAPA has potentiating effects on CD4^+^CD25^+^ Treg [16], [22], [23], [25]. The notion that RAPA may induce tolerance via expansion of Treg numbers and enhancement of their function has introduced a novel paradigm into RAPA activity as an immunosuppressive drug.

In the present work, we demonstrate that in human PBMC obtained from normal donors, RAPA selectively promotes activation and expansion of highly suppressive CD4^+^CD25^high^Foxp3^+^ Treg cells. These cells are resistant to apoptosis, while activated conventional CD4^+^CD25^neg^ T cells become highly sensitive to RAPA, which has been reported to block the survival PI3K/Akt/mTOR pathway these cells preferentially utilize [31]. Thus, responses to activation-induced cell death of human Treg and Tconv appear to be divergently regulated in the presence of RAPA. The CD4^+^CD25^high^ Treg up-regulate expression of the anti-apoptotic Bcl-2 family members (i.e., Bcl-2 and Bcl-xL) in the presence of RAPA, while the conventional CD4^+^CD25^neg^ T cells significantly down-regulate expression of Bcl-2 or Bcl-xL increasing the relative levels of pro-apoptotic Bax and thus promoting apoptosis of this subset of T cells. The resulting shift in the Bcl-2/Bax or Bcl-xL/Bax ratio favoring anti-apoptotic proteins in the CD4^+^CD25^high^ T cells cultured with RAPA could be responsible for resistance of Treg to apoptosis. Thus, our results provide a rationale for the use of RAPA in order to expand human Treg that are resistant to apoptosis and have potent suppressor functions. Earlier data in mice have also suggested that RAPA might be used *in vivo* to expand Treg subsets, while depleting the excess of conventional T cells [31]. The definition of optimal conditions for RAPA-driven Treg proliferation is an important objective, because Treg are a minor component of peripheral blood lymphocytes. The ability to reproducibly generate cultures of pure, well-defined and highly suppressive Treg for adoptive transfers to patients with autoimmune diseases or after transplantation in the future remains a challenge. While Treg expansion from cord blood has been previously described [18], [20], [21] and is currently used for therapy to prevent post-transplant graft versus host disease (GVHD), transfers of autologous, adult blood-derived Treg are preferable in today's regulatory climate and also more feasible.

Expression of CD25 (IL-2Rα) on human nTreg suggest that these cells are dependent on IL-2 for proliferation and survival. Signaling via CD25 is crucial for the Treg development and function [31], and no Treg are present in mice that are IL-2 deficient [33], [34], IL-2Rα-deficient [34] or IL-2Rβ-deficient [35]. Further, IL-2 directly regulates Foxp3 expression and proliferation of human Treg via the STAT pathway [36]. Similarly, cytokine production and proliferation of murine Treg has been shown to be mainly STAT5-dependent [37]. In T cells, signaling downstream of the IL-2R can proceed via the PI3K/Akt/mTOR or JAK/STAT pathways. While RAPA inhibits the mTOR/PI3K pathway in Treg, JAK/STAT signaling is not affected, sparing Treg from RAPA-induced apoptosis [38]. The significant role of Akt-mTOR pathway in regulating de novo differentiation of CD4^+^FOXP3^+^ Treg has been emphasized by Haxhinasto et al [39]. Battaglia and colleagues have speculated that RAPA may have an inhibitory effect on IL-2 production by activated Tconv, and that Treg able to effectively compete for IL-2 are favored to expand in the presence of RAPA [40]–[42]. While murine experiments indicate that IL-2 is required for expansion of Treg, we have shown that IL-2 up-regulates expression of Bcl-2 and Foxp3 in human Treg and that Treg suppressor functions depend on the IL-2 concentration in their microenvironment [43]. Our data demonstrating that RAPA enriches for CD4^+^CD25^high^ T cells with a high expression level of Foxp3 are consistent with this paradigm. Expansion of IL-2 and TCR-activated Treg is regulated by STAT5. The Foxp3 promoter has been shown to contain consensus sequences which bind STAT5, resulting in high levels of STAT5 expression in Treg [36]. Interestingly, STAT3 signaling also appears to be up-regulated in CD3^+^CD25^high^ cells by RAPA. In aggregate, these *in vitro* results with human Treg support a large body of murine data which indicate that in Treg, the STAT signalling pathway is preferentially utilized over the PI3K pathway.

PTEN is an oncogene involved in regulation of a broad variety of cellular functions, including cell proliferation and cell death [31]. PTEN is a negative regulator of the mTOR pathway, and it inhibits IL-2R-mediated expansion of CD4^+^CD25^+^ Treg [44]. Elevated PTEN levels observed in human Treg suggest that these cells have a reduced usage of the PI3K/mTOR pathway, in contrast to CD4^+^CD25^neg^ Tconv, which depend on the PI3K pathway for their proliferation and survival. If PTEN blocks downstream events in the PI3K pathway in CD4^+^CD25^high^ T cells, then the reduced expression of phosphorylated mTOR and phosphorylated 4E-BP1 and p70S6K1 proteins should be evident in these cells relative to CD4^+^CD25^neg^ Tconv. Indeed, our results confirm this hypothesis and suggest that Treg depended on the IL-2R-mediated JAK/STAT signaling for proliferation, so that it is not inhibited by RAPA. In contrast, CD4^+^CD25^neg^ Tconv responding to TCR and IL-2 stimulation signals via the PI3K pathway are sensitive to RAPA-induced inhibition of proliferation. Recent data of Buckler at al in mice suggest that by negatively regulating TCR-mediated signals, PTEN imposes a requirement for CD28 co-stimulation to assure T-cell proliferation and survival [45]. Their observation together with our finding that PTEN is highly expressed in human CD4^+^CD25^high^ Treg might explain why human postthymic Treg expanded *in vitro* in the presence of RAPA require CD28 co-stimulation for survival and suppressor function *in vivo* [46]. In this context, the CD28-PTEN pathway might represent a novel regulation mechanism of peripheral tolerance mediated by Treg.

The observations we report for human Treg endorse the molecular models of Treg signaling proposed for murine T cells [25], [30]. As Treg play a critical role in control of autoimmune diseases, transplant rejection and cancer, a better understanding of molecular signaling they utilize is essential for improving therapeutic interventions in these diseases. While the precise molecular mechanisms operating the survival or death pathways in T cells are still under investigations, the possibility of their pharmacologic manipulation in the future might open the way for the development of new immunotherapeutic strategies that will permit the “long-term” induction and selective expansion of Treg or provide clues about their selective elimination. The permanent depletion of Treg in patients with cancer or infectious diseases might be beneficial, allowing for expansion of T effector and helper T cells and circumventing the side effects of current therapies which cause general immunosuppression by depleting all activated T cells.

## Materials and Methods

### Media and reagents

All cell culture reagents, including AIM V, DMEM, RPMI 1640 media, phosphate-buffered saline (PBS), streptomycin (S), penicillin (P), sodium pyruvate L-glutamine, non-essential amino acids, and trypan blue dye were purchased from Gibco/Invitrogen, Grand Island, NY. Culture media, were supplemented with pre-tested human AB serum (8%, v/v) purchased from Geminie Bioproducts (West Sacramento, CA), penicillin (100 IU/mL), streptomycin (100 μg/mL) and L-glutamine (2 mM/L) and cell cultures were incubated at 37°C in an atmosphere of 5% CO_2_ in air. Recombinant human IL-2 (rh IL-2) was purchased from Peprotech, Rocky Hill, NJ. Anti-CD3 mAb (OKT3) was obtained from ATCC, Rockville, MD. Anti-CD3 and anti-CD28 mAb-coated beads were from Beckman Coulter, Miami, FL. Rapamycin (RAPA), saponin and Brefeldin A were from Sigma, St. Louis, MO. Recombinant human (rh) IL-6 was purchased from Cellgenix, Antioch, IL.

### Antibodies for flow cytometry

The following anti-human mAbs were used for flow cytometry analysis: anti-CD3-ECD, anti-CD4-PC5, anti-CD25-FITC and -PE, anti-CD122-FITC, anti-CD132-PE, anti-CD45RO-FITC, anti-Fas-FITC, anti-IFNγ-FITC, anti-CD62L-FITC and anti-CD152-PE were purchased from Beckman Coulter, (Miami, FL); anti-h/TNFRSF18-GITR (clone FAB689F) and anti-CCR7-FITC from R&D Systems, Inc., (Minneapolis, MN); anti-TGF-β_1_ (unconjugated) was from Antigenix America, Inc., (Huntington Station, NY); anti-Foxp3-FITC (clone PCH101); anti-IL-4-FITC and anti-CD127-FITC from eBioscience (San Diego, CA); anti-FasL-PE (NOK-1.42 kDa) and anti-Perforin-FITC from Biolegend (San Diego, CA); anti-Granzyme B-PE (clone GB111) from PeliCluster Inc, (Netherlands) and anti-Granzyme A-PE, anti-CD45RA-FITC, anti-IL-10-PE and isotype controls IgG_1_, IgG_2a_ and IgG_2b_ were all purchased from BD Pharmingen, (San Jose, CA). The following antibodies were used to study the anti- or pro-apoptotic pathways involved in responses to rapamycin: anti-Bcl2-FITC, anti-Bcl-xL-FITC, anti-Bax-FITC (Santa Cruz Biotechnology Inc., CA), anti-Bid (unconjugated; Abcam, Cambridge, MA) and anti-FLIP (unconjugated; Genway Biotech, Inc., San Diego, CA. Additional Abs used for flow cytometry were all purchased from Cell Signaling, Danvers, MA and included: anti-PTEN-Alexa Fluor 488, anti-phospho-4E-BP1-Alexa Fluor 488 and anti-phospho-p70S6 Kinase polyclonal, unconjugated used with FITC-donkey anti-rabbit IgG (Jackson Immuno Research Laboratories) as a secondary Ab. Prior to use, all mAbs were titrated using normal resting or activated PBMC to establish optimal staining dilutions.

### Cell isolation and separation

Peripheral blood mononuclear cells (PBMCs) were isolated from buffy coats obtained from 21 normal donors (Central Blood Bank of Pittsburgh) on Ficoll-Hypaque density gradients (GE Healthcare Bio-Sciences Corp., Piscataway, NJ). Cells recovered from the gradient interface were washed twice in RPMI 1640 medium, counted and separated into monocytes and lymphocytes via plastic adherence. The non-adherent fraction was immediately used for magnetic cell isolation using AutoMACS (Miltenyi Biotec, Auburn, CA) as previously described [16]. CD4^+^ T cells were negatively selected with a CD4^+^ T-cell isolation kit (Miltenyi Biotec, Auburn, CA), yielding populations of CD4^+^ cells with the 96–99% purity. Next, CD4^+^CD25^+^ T cells were separated from CD4^+^CD25^neg^ T cells on the AutoMACS in two repetitive separation steps using CD4^+^CD25^+^ T Regulatory Cell Isolation Kit (Milltenyi Biotec). Positively selected CD4^+^CD25^+^ T cells and negatively depleted CD4^+^CD25^neg^ T cell fractions (purity >98%) were immediately used for expansion protocols or suppression assays. Aliquots of the CD4^+^CD25^neg^ cells were cryopreserved to be used as autologous responder cells in suppression assays.

### Expansion of human CD4^+^CD25^+^ Treg

Using a previously developed protocol [16], we cultured MACS-isolated T cells in the presence of anti-CD3/CD28 Ab-coated beads and IL-2 [16], [27]. Briefly, purified CD4^+^CD25^+^ or CD4^+^CD25^neg^ T cells (1×10^5^ cells/well) were initially cultured in 96-well plates in AIM V medium supplemented with soluble OKT3 (1 μg/mL), IL-2 (150 IU/mL) and irradiated (3,000R) autologous feeder cells (2 feeder cells to 1 Treg) for 5 to 7 d. Next, the cells were transferred to wells of a 24-well plates (at least 2.5 to 3×10^5^ cells/well) and re-stimulated with anti-CD3/CD28 mAb-coated beads (1 viable cell to 4 beads). On the 3rd day, IL-2 (1,000 IU/mL) was added and the cells were cultured for 7–8 days. On Day 8, the cells were re-stimulated with anti-CD3/CD28 mAb and IL-2 (1,000 IU/mL) and RAPA at the final concentration of 1, 10, 50 or 100 nM was added to half of the cultures. Cultures were supplemented with fresh medium plus RAPA as needed. After 3 weeks of expansion in the presence of RAPA the beads were removed using a magnet and the cells counted. Cells (at least 6×10^5^ cells/well) were transferred to wells of 6-well plates and again re-stimulated with anti-CD3/CD28 mAb-coated beads and 1,000 IU/mL IL-2 in the presence or absence of RAPA for 1-2 weeks. On the final day of culture anti-CD3/CD28 mAb-coated beads were removed, cells were washed in medium, counted in the presence of a trypan blue dye and resuspended in medium containing 50 IU/mL of IL-2 for 24–48 h prior to all phenotypic or functional assays. Expanding T cells were routinely analyzed at 3 different time points: at weeks 1, 3 and 6. **R0** are cultures of CD4^+^CD25^+^ or CD4^+^CD25^neg^ T cells in the absence of RAPA, while+**R** are cultures of these T cell subsets+RAPA.

### Surface and intracellular staining

After harvest, cells were washed and stained with the above listed Abs for 15 min at 4°C as previously described [16], [28]. Appropriate isotype control Abs were used for each sample. Following staining, cells were examined by four-color flow cytometry.

Intracellular staining was performed as previously described [16]. Expression levels of TGF-β_1_, IL-10, Foxp3, CTLA-4, BcL-2, Bax, BcL-xL, PTEN, 4E-PB1 and p70S6K1 were assessed before and after activation of cells with anti-CD3/CD28 beads and IL-2 (±RAPA). Briefly, samples were first incubated with mAbs against surface markers. After washing, cells were fixed with 4% (v/v) paraformaldehyde in PBS for 20 min at RT, washed once with flow solution (PBS containing 0.5% (v/v) BSA and 2 nM EDTA), permeabilized with PBS containing 0.5% BSA (v/v) and 0.1% (v/v) saponin and stained with detection Abs specific for intracytoplasmic proteins for 30 min at RT. Cells were further washed twice with PBS containing 0.5% BSA (v/v) and 0.2% (v/v) saponin, fixed with 4% (w/v) paraformaldehyde and analyzed by flow cytometry. Appropriate isotype controls were included for each sample. Cells stained with the primary unconjugated Ab were incubated with the secondary Ab (anti-rabbit-FITC-IgG, 1500 ng/mL) purchased from Jackson ImmunoResearch Laboratories Inc., West Grove, PA, or with a control secondary Ab (anti-mouse-FITC-IgG at 1∶10 dilution; Beckman Coulter) in saponin for 15 min, washed twice with PBS containing 0.5% BSA and 0.2% (v/v) saponin and analyzed by flow cytometry.

### Suppression assays

Responder CD4^+^CD25^−^ T cells (RC) were autologous to suppressor cells (S). Freshly-isolated RC were cryopreserved. Prior to their use in suppression assays, RC were thawed and maintained in medium plus 50 IU/mL of IL-2 for 48 h. RC were stained with 1.5 μM CFSE (Molecular Probes/Invitrogen, Carlsbad, CA) as previously described [16] and co-cultured with S in a complete AIM V medium containing IL-2 (150 IU/mL), plate-bound OKT3 (1 μg/mL) and soluble anti-CD28 Ab (1 μg/mL) in wells of 96-well plates (at least 5×10^5^/well). S were CD4^+^CD25^high^ or CD4^+^CD25^neg^ T cells expanded±RAPA under conditions described above for 3–6 weeks and added to RC at the S∶R ratios of 1∶1, 1∶5 and 1∶10. Co-cultures were incubated at 37°C in an atmosphere of 5% CO_2_ in air for 3–5 d. After harvest, suppression of CFSE-labeled RC proliferation was analyzed. Data obtained by flow cytometry were analyzed as previously described by us [3], [5], [16] using ModFit LT for Win32 software provided by Verity Software House, Inc., Topsham, ME. The percentages of suppression were calculated based on the proliferation index (PI) of RC alone compared with the PI of cultures containing RC and Treg. The program determines the percent of cells within each peak, and the sum of all peaks in the control culture is taken as 100% of proliferation and 0% of suppression.

### Annexin V binding

The binding of ANX V-FITC (Apoptosis Detection Kits, BD PharMingen) to phos-phatidylserine on T-cell surface was used as a measure of apoptosis. Stained samples were analyzed by flow cytometry. The gates were set to include the live (PI-) as well as the dead (PI+) cells present in each sample.

### Expression of phosphorylated STAT3/5 proteins

Expression of phosphorylated STAT3 and STAT5 in CD4^+^CD25^high^ Treg and conventional (CD4^+^CD25^neg^) T cells was determined in freshly purified T cell subsets following a brief (20 min) stimulation with 150 IU/mL IL-2 (STAT5-expression) or 150 IU/mL IL-6 (STAT3-expression). Cells were cultured±RAPA for a short (4 hr) or longer time periods (1, 2, 3 or 4 weeks), harvested, washed, stimulated with the cytokines for 20 min and then stained for analysis with anti-CD3-ECD, anti-CD4-PC5 and anti-CD25-PE Abs followed by intracellular staining with the anti-phosphorylated STAT5 Ab (pY694) labeled with Alexa Fluor-488 or the Ab specific for phosphorylated STAT3 Ab (pY705)-Alexa Fluor-488 (both from BD Biosciences). The gate for flow cytometry was set on CD3^+^CD4^+^ cells and then on CD25^neg^ or CD25^high^ cells.

### Western blot analysis

Cells (CD4^+^CD25^+^ and CD4^+^CD25^neg^) were lysed at 4°C at the concentration of 0.5×10^6^ cells/mL. The lysis buffer, SDS-PAGE electrophoresis and blotting conditions were as previously described [29]. Membranes were washed and probed with the following polyclonal Abs, all purchased from Cell Signaling: phospho-p70S6Kinase (Thr 389); phospho-4E-BP1 (Thr 37/47); mTOR (7Clo) rabbit mAb; phospho-mTOR (Ser 2448); phospho-mTOR (Ser 2481); phospho-PI3Kp85 (Tyr 458)/p55. Blots were visualized by ECL (Roche Diagnostics, Indianapolis, IN) according to the manufacturer's protocol. For reprobing, Abs were first stripped using Restore Western Blot stripping buffer (Pierce, Rockford, IL) and then the membranes were blotted with the second Ab.

### Statistical Analysis

The arithmetic means and SD values were calculated for all parameters in at least ten independent experiments. Statistical analysis of differences in all of the data was done using the one-way ANOVA test. P values <0.05 were considered significant.

## Supporting Information

Figure S1 (1.91 MB TIF)
