# Supplementary figures and images for: Differential Responses of Human Regulatory T Cells (Treg) and Effector T Cells to Rapamycin
Source: PLoS One. 2009 Jun 22;4(6):e5994. doi: 10.1371/journal.pone.0005994 (PMC2694984; doi:10.1371/journal.pone.0005994)

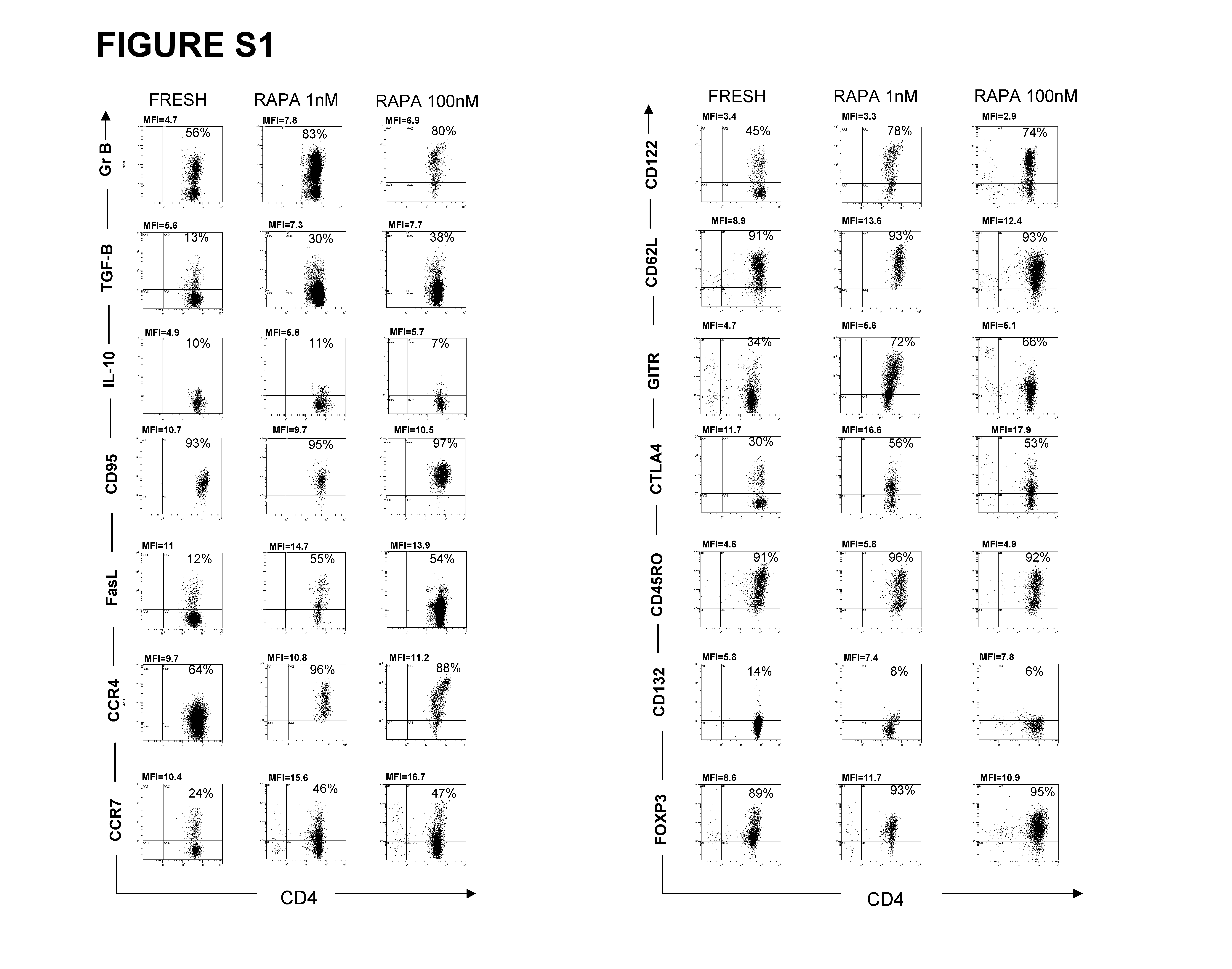

Supplement: Figure S1 — (1.91 MB TIF) [file pone.0005994.s001.tif]
